# Risk factors affecting patients survival with colorectal cancer in Morocco : Survival Analysis using an Interpretable Machine Learning Approach

**DOI:** 10.21203/rs.3.rs-2435106/v1

**Published:** 2023-01-10

**Authors:** Imad El Badisy, Zineb BenBrahim, Mohamed Khalis, Soukaina Elansari, Youssef ElHitmi, Fouad Abbas, Nawfal Mellas, Karima EL Rhazi

**Affiliations:** 1 Mohammed VI Center for Research & Innovation, Rabat, Morocco. Mohammed VI University of Health Sciences (UM6SS), Casablanca, Morocco; 2 INSERM, IRD, SESSTIM, Sciences Economiques & Sociales de la Santé & Traitement de l’Information Médicale, Aix Marseille Univ, Marseille, France; 3 Department of Oncology, University Hospital Hassan II, Sidi Mohamed Ben Abdellah University, Fez, Morocco; 4 Department of Epidemiology and Public Health, Faculty of Medicine of Fez, Research Laboratory “Epidemiology and Research in Health Sciences”, Sidi Mohamed Ben Abdillah University, Fez, Morocco

**Keywords:** colorectal cancer, prognostic factors, overall survival, cox model, random survival forest, variable importance, partial dependance plots

## Abstract

The aim of our study was to assess the overall survival rates for colorectal patients in Morocco and to identify strong prognostic factors using a novel approach combining survival random forest and the Cox model.

Covariate selection was performed using the variable importance based on permutation and partial dependence plots were displayed to explore in depth the relationship between the estimated partial effect of a given predictor and survival rates. The predictive performance was measured by two metrics, the Concordance Index (C-index) and the Brier Score (BS).

Overall survival rates at 1, 2 and 3 years were, respectively, 87% (SE = 0.02; CI-95% = 0.84–0.91), 77% (SE = 0.02; CI-95% = 0.73–0.82) and 60% (SE = 0.03; CI-95% = 0.54–0.66). In the Cox model after adjustment for all covariates, sex, tumor differentiation had no significant effect on prognosis, but rather tumor site had a significant effect. The variable importance obtained from RSF strengthens that surgery, stage, insurance, residency, and age were the most important prognostic factors. The discriminative capacity of the Cox PH and RSF was, respectively, 0.771 and 0.798 for the C-index, while the accuracy of the Cox PH and RSF was, respectively, 0.257 and 0.207 for the Brier Score. This shows that RSF had both better discriminative capacity and predictive accuracy.

Our results show that patients who are older than 70, living in rural areas, without health insurance, at a distant stage and who have not had surgery constitute a subgroup of patients with poor prognosis.

## Introduction

Colorectal cancer is the 3rd most diagnosed cancer in the world, with more than 1.931 million new cases in 2020 worldwide and 4558 new cases in Morocco, representing almost 7.7 percent of all new cancer cases in the country. Several factors are significantly associated with the prognosis of CRC patients [28, 2, 32]. Whether these factors are related to patient characteristics, treatments, or even the healthcare system in general, it is essential to study their effects in order to develop a care strategy adapted to the local context.

Moreover, CRC imposes an economic burden that translates into direct medical expenses (cost of screening, hospitalization, treatment, transportation, etc.) and indirect expenses such as loss of productivity [4, 42].

Financial and insurance barriers have a major effect on the survival of patients with cancer. They are generally identified as directly related to health care utilization. For example, financial concerns have been found to prevent uninsured people from seeking care unless they were in severe pain or thought they were going to die [3, 9]. In a study on CRC patients, 18% reported untreated rectal bleeding and 20% reported a change in bowel habit but never sought medical attention. The main reason was that they did not consider this symptom to be serious [11].

Furthermore, there is considerable epidemiological and observational evidence that the risk of colorectal cancer is closely related to lifestyle, particularly diet and physical activity [31, 7, 36].

The aim of our study was to assess the overall survival rates for CRC at 3 years and to identify associated strong prognostic factors among patients in Morocco using a novel approach combining survival random forest and the Cox PH model.

## Methods

### Study design and data collection

We performed a retrospective analysis of 343 patients diagnosed and followed at Hassan II University Hospital. Data about patients’ characteristics was extracted from the patients’ medical records and supplemented with an active follow-up to record vital status and observed survival times. Eligible patients had a histologically confirmed diagnosis of colorectal cancer. Patients with histological types other than adenocarcinoma (N = 200) and diffuse lattice type (N = 50) were excluded. Similarly, patients with uninformed medical records were removed (N = 150).

For each patient, information on sex, age, insurance, residency, delay to treatment, personal history, tumor site, stage, tumor differentiation, histological type, surgery, and MSI/MSS status, was extracted.

The date of diagnosis of colorectal cancer indicated the start of the observation period. Patients were followed from January 2009 to January 2015 until death or censored at the end of the study. The end-point was set at 36 months from the date of diagnosis.

### Ethical considerations

This study was approved by the Ethics Committee of the Hassan II University Hospital of Fes under the reference n° 05/18. Due to the retrospective nature of this sutdy and that only medical history data were collected, informed consent was waived by the aforementioned Ethics Comittee. All methods in this study were carried out in accordance with relevant guidelines and regulations.

### Informed consent is waived since retrospective nature of study by ( name of waiving committee who have waived the consent)

#### Statistical analysis

A descriptive analysis of the study sample was conducted. The quantitative variables are presented as mean, median, minimum, and maximum, while the categorical variables are presented as numbers and proportions.

Imputation of missing data was done using the missRanger algorithm. It implements an imputation approach based on the random forest algorithm combined with the predictive mean matching method [33, 44]. This is a non-parametric imputation method that makes no prior assumptions about the distribution of the data. It directly predicts missing values using a random forest trained on the observed parts of the data set. The imputation is performed iteratively until a convergence criterion is reached.

Overall survival rates (1, 2 and 3 years) and corresponding 95% CIs were calculated using the nonparametric Kaplan-Meier estimator [26]. Statistical comparison of the survival curves was performed using the log-rank test when stratification was performed on categorical variables.

The Cox Proportional Hazards (Cox PH) model and Random Survival Forests (RSF) were both used to identify factors that affect CRC patient survival in our study. The well-known Cox PH regression model was used to estimate the effect of prognostic factors on survival time. The Multivariate Cox PH model with all the potential baseline predictors was estimated in order to compute the hazard ratio (HR) and their associated 95% Cis [12]. Baseline predictors were: sex, age, insurance, residency, delay to treatment, personal history, tumor site, stage, tumor differentiation, histological type, surgery, and MSI/MSS status.

Beyond the Kaplan-Meier estimator, the Cox PH model allows the inclusion of covariates, which is useful for refining the information on survival time. In this case, the statistical significance of the adjusted covariates on survival times is tested.

The Cox PH is a semi-parametric model with two components, one parametric related to predictor variables and another fully nonparametric related to the estimate of the survival function, which doesn’t make any assumptions about the underlying distribution of survival times.

In practice, the Cox model is specified by a hazard function:

$$h(t)=h_{0}(t)exp\left(\beta^{T}X_{i}\right)$$

where *h*_0_(*t*) is the risk function (i.e. instantaneous chance at inclusion). This risk function is modified by changes in survival time conditional on covariates, *X*_*i*_ = *X*_1_, . . . , *X*_*p*_ is a vector of covariates that do not depend on time, and *β*_*i*_ = *β*_1_, . . . , *β*_*p*_ is a vector of regression coefficients associated to *X*_*i*_. The parameters of the vector *β*_*i*_ are estimated by maximizing the partial true likelihood expressed as follows:

$$L(\beta )=\prod_{i=1}^{m}\frac{exp\left(\beta^{T}X_{i}\right)}{\sum_{j\in R_{i}}exp\;\left(\beta^{T}X_{i}\right)}$$

where *R*_*i*_ is the set of subjects at risk at time *t*_*i*_, either *y*_*i*_ ≥ *t*_*i*_.

Several approaches can be used for model selection. A simple method is to estimate a univariate regression model, then a multivariate model with all predictors statistically significant (p-value > *α*, *α* = 0.05). However, this univariate statistical significance filtering approach does not take into account interactions between covariates.

#### Random Survival Forests

In order to identify the most influential predictors of survival outcome, we extended our data analysis by performing a Random Survival Forest. The RSF is an extension of the classical random forest framework to right-censored observations [22, 6].

The main advantage of this approach is that it does not require any restrictive assumptions on the distribution of the data, unlike the proportional hazard assumption for the Cox model [35]. As a first step, binary survival trees are developed using the bootstrap sampling procedure for all predictors included in the analysis, by recursive partitioning similar to CART [13]. Each bootstrap sample excludes about 37% of the out-of-bag (OOB) data used as an estimate of the predictive error. Then the log-rank test statistic is specified as the default dump rule for splitting survival trees [10].

The final forest set is calculated by averaging the end node statistics using the boosted Nelson-Aalen and Kaplan-Meier estimators [22].

#### Variable importance

Another approach to covariate selection is the variable importance method (VIMP) based on permutation. With this method, the attributable prediction error of each predictor *X*_*i*_ is calculated. This approach is defined by [16] as the difference in model predictive performance between datasets with and without permuted values for the associated variable.

Permutation-based variable importance as implemented in SRF permutes the OOB data of a variable and compares its OOB prediction error with the original one. The intuition behind this method is that large importance values indicate variables with strong predictive potential [6].

#### Partial Dependence Plots

Finally, partial dependence plots were displayed to explore in depth the relationship between the estimated partial effect of a given predictor and survival rates [22].

PDP can reveal the shape of the relationship between a covariate and the target variable. Its values are constructed by drawing a subset of patients at random and then predicting their survival with the random forest many times, while holding all the covariates constant except for the covariate for which we want to estimate its marginal effect. This gives risk curves for each individual in the subset, normalized by its mean to obtain a single risk curve. For categorical covariates, PDP gives the risk associated with a certain class given different values of the covariate. Only the final set of strong predictors obtained by the VIMP procedure was considered in our interpretation.

#### Predictive accuracy

Finally, we evaluated the predictive performance of our two models, Cox PH and RSF. The predictive performance was measured by two metrics, the Concordance Index (C-index) and the Brier Score (BS). The C-index is the frequency of concordant pairs among all pairs of subjects. It can be used to assess and compare the discriminative power of a risk prediction survival model [19]. A pair of patients (*i*, *j*) is called concordant if the risk of the predicted event by the model is lower for the patient who experiences event at a later time point.

$$\text{C-index}\;=\frac{\sum_{i,j}1_{T_{j}<T_{i}}\;\cdot \;1_{\eta_{j}>\eta_{i}}\;\cdot \;\delta_{j}}{\sum_{i,j}1_{T_{j}<T_{i}}\;\cdot \;\delta_{j}}$$

with *η*_*i*_ the risk score of a unit *i* and $1_{T_{j}<T_{i}}=1$ if *T*_*j*_ *< T*_*i*_ else 0 and $1_{\eta_{j}>\eta_{i}}=1$ if *η*_*j*_ *> η*_*i*_ else 0. C-index takes values between 0 and 1, with 1 corresponding to the best discriminative power of the model.

The Brier score is used to evaluate the accuracy of a predicted survival function given a vector of time *t*. This is an improved version of the prediction error at the time point using inverse probability weighting of the censoring [18].

$$BS(t)=\frac{1}{N}\sum_{i=1}^{N}\left[\frac{{\left(\hat{S}\left(t\;\vee \;z_{i}\right)\right)}^{2}}{\hat{G}\left(X_{i}\right)}\cdot I\left(X_{i}<t,\;\delta_{i}=1\right)+\frac{{\left(1\;-\;\hat{S}\left(t\;\vee \;z_{i}\right)\right)}^{2}}{\hat{G}(t)}\cdot I\left(X_{i}\geq t\right)\right)$$

Where *t* is the time point at which BS is calculated, *N* is the sample size, *x*_*i*_ is the covariate corresponding to sample *i*; *S*^(·)^ is the survival function predicted by the model and ^(·)^ is the survival function corresponding to censoring. BS take values between 0 and 1, with 0 the best possible value corresponding to the best predictive accuracy of the model.

All statistical analyses were performed using R [40]. Specifically, Kaplan-Meier curves and cox models were computed using the survival package [47]. The finalfit package [20] was used to display the univariate and multivariate cox regression tables. The survminer package [27] was used to draw the KM curves. The missRanger package [33] was used to impute missing values from the original data set. The RandomForestSRC package [23] was used to build random survival forests, perform variable importance and display partial dependence plots.

## Results

A total of 346 patients were included for analysis. Details of demographic characteristics are described in Table 1. Overall, 181 were female (52.31%), with a mean age of 56.5 years (SD = 13.4).

Table 2 describes the clinical characteristics of CRC patients. There was almost an equal proportion of patients with colon (N = 186, 53%) and rectum (N = 160, 47%) cancers. The left colon was the most common tumor subsite (143 , 41.3%), followed by the inferior (84, 24.3%) and middle rectum (58, 16.8%). More than half of the patients were at a distant stage at the time of inclusion (189, 54.6%). Furthermore, the predominant histological type was mucinous (313, 90.5%).

Table 3 presents the overall survival rates at 1, 2 and 3 years, which are, respectively, 87% (SE = 0.02; CI-95% = 0.84–0.91), 77% (SE = 0.02; CI-95% = 0.73–0.82) and 60% (SE = 0.03; CI-95% = 0.54–0.66).

**Figure 1 :**
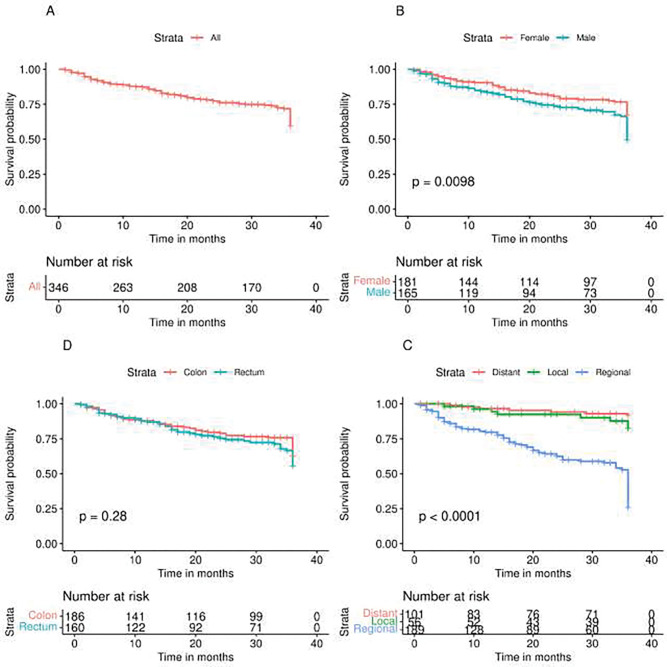
Kaplan-Meir survival curves

The difference in the survival curve was statistically significant by sex (log-rank test, p < 0.0098). The difference was also statistically significant when the survival curve was stratified by stage (log-rank test, p = 0.0001). However, no statistical significant difference was observed when stratifying by site (log-rank test, p = 0.28) (Figure 1).

Only MSI/MSS status, Delay and histological type had missing values, respectively, 4.6%, 2.89% and 0.28%.

### Univariate and multivariate Cox proportional hazard

The survival probability of CRC patients depends on a variety of complex factors. In the univariate Cox PH regression, covariates such as sex, age, insurance, residency, stage, and surgery showed a significant prognosis effect on the overall survival.

While in the multivariate analysis after adjustment for all covariates, sex, tumor differentiation had no significant effect on prognosis, but rather tumor site had a significant effect.

The results of the multivariate Cox PH model illustrate that patients who did not have surgery were 3.21 times more likely to die (HR = 3.21; CI = 1.83–5.63; p < 0.001) than those who did, and those with a distant stage were 6.64 times more likely to die (HR = 6.64; CI = 2.80–15.72; p < 0.001). Patients with no health insurance had 2.85 times a higher risk of mortality (HR = 2.85; CI = 1.63–4.98; p < 0.001) than patients with health insurance. Similarly, patients living in rural areas had a 1.88 times greater risk of death (HR = 1.88; CI = 1.18–2.98; p < 0.001) compared to those living in urban areas. Besides, patients with a tumor in the rectum side had a 1.86 times greater risk of death (CI = 1.21; 2.88; p = 0.005) than patients with a tumor in the colon side.

After the sensitivity analysis performed by RSF, we decided to keep all predictors in the multivariate Cox PH model in order to preserve the interaction between covariates, while interpreting covariates that have a statistically significant effect in the Cox model, and their effect is confirmed by the RSF.

### RSF variable importance

The RSF was fitted using the same covariates as in the Cox PH models. A variable importance permutation-based approach was used to identify the most important prognostic factors related to the overall survival of our group of patients, and confidence intervals were calculated using subsampling.

**Figure 2 :**
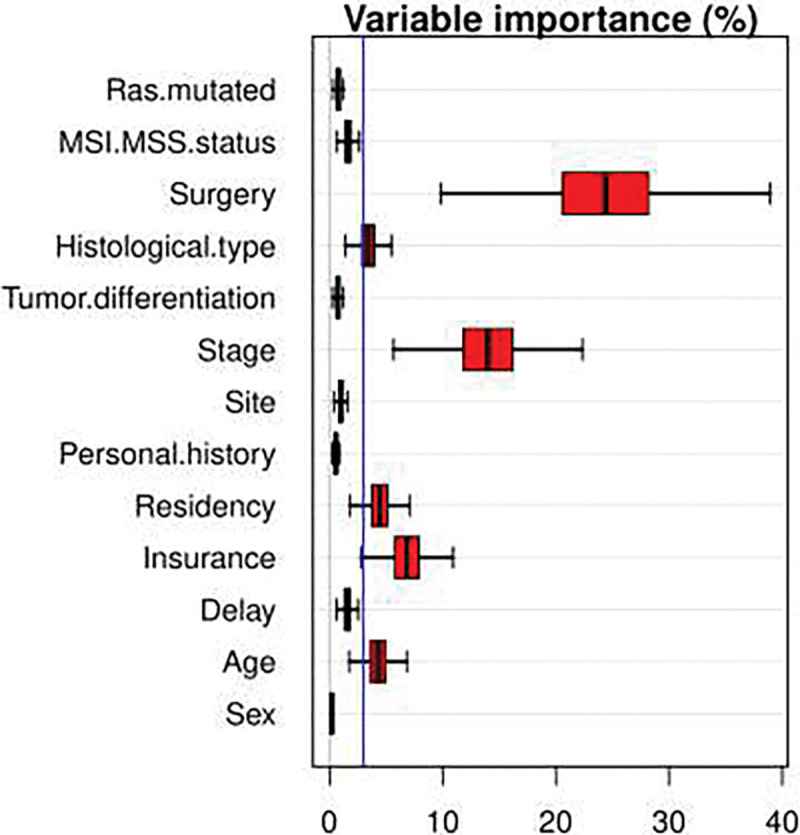
Variable importance of random forest through permutation

The variable importance obtained from RSF strengthens that surgery, stage, insurance, residency, and age were the most important prognostic factors (Figure 2).

These results are in agreement with the results obtained from fitting the multivariate Cox PH model (Table 4), as far as statistical significant effects are concerned.

**Figure 3 :**
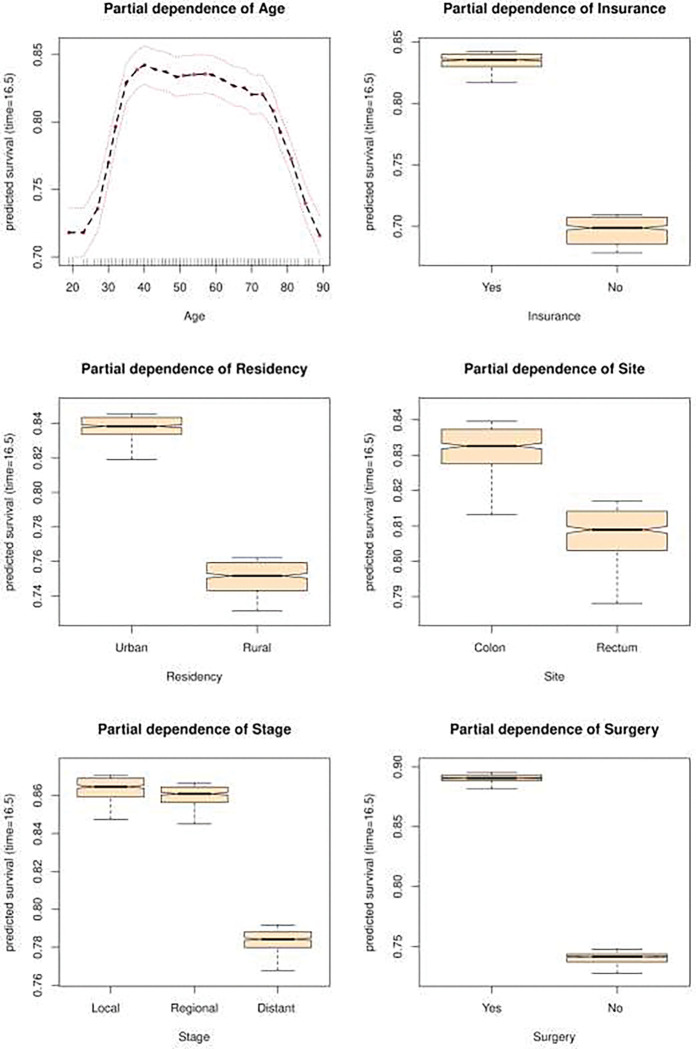
Partial dependence plots

Figure 3 depicts Partial Dependence Plots. For age, PDP shows that the survival rate increases from 18 to 30 years, decreases very slowly between 30 and 75 years, and drops after that age. For insurance, we observed a positive effect for the ‘Yes’ modality compared to the ‘No’ modality on the survival rate, with a relative difference of almost 15 percentage points. For residency, we observed a positive effect on the survival rate for the ‘Urban’ modality compared to the ‘Rural’ modality, with a relative difference of almost 9 percentage points. For the site, a positive effect on the survival rate for the ‘Colon’ modality compared to the ‘Rectum’ modality was shown, with a relative difference of almost 3 percentage points. Furthermore, the ‘Distant’ stage had a negative effect on the survival rate when compared to the ‘Local’ stage, with a relative difference of nearly 7 percentage points. For surgery, the ‘Yes’ modality had a positive effect on the survival rate compared to the ‘No’ modality, with a relative difference of almost 15 percentage points.

### Predictive performance

**Figure 4 :**
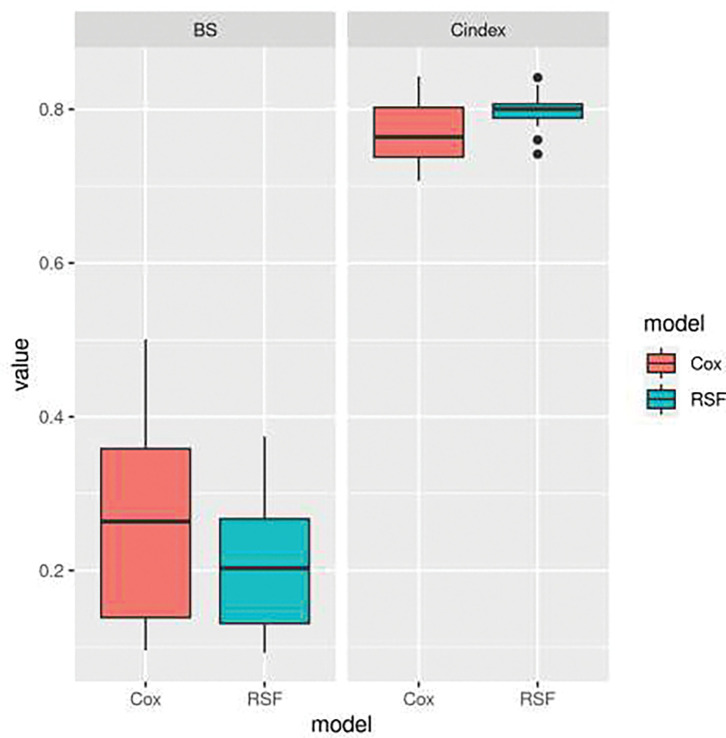
Predictive performance using C-index and IBS

The predictive performance of the Cox PH and SRF was evaluated using the Concordance Index and the Integrated Brier Score. The discriminative capacity of the Cox PH and RSF was, respectively, 0.771 and 0.798 for the C-index. while the accuracy of the Cox PH and RSF were, respectively, 0.257 and 0.207 for the Brier Score. This shows that RSF had both a better discriminative capaciy and predictive accuracy (Figure 4).

## Discussion

Colorectal cancer (CRC) is one of the most common cancers diagnosed globally. However, due to earlier detection and more effective treatment, high-income countries have seen a significant decrease in CRC mortality over the past decades. Personalizing treatment, focusing on high-risk patients, and improving access to health-care systems are critical to achieving the best possible health outcome.

In Morocco, there are two Population-Based Cancer Registries (PBCR): one in Casablanca that covers about 12% of the national population (36 million people) and has 38 data sources, and another in Rabat that covers about 21% of the national population (642000 people) and has 65 data sources [5, 46]. But as it is known, PBCR make a trade-off between exhaustivity in terms of incidence and availability of variables about patients (i.e., prognostic factors). Therefore, the study of factors influencing survival times is a difficult exercise with this type of data. Nevertheless, we have attempted for the first time in Morocco to study the survival of CRC and associate it with both socio-demographic factors and histopathological characteristics.

We found only one Moroccan study in the literature on the effect of predictive factors, particularly surgery, on survival of patients with mid and low rectal aenocarcinoma [14]. The overall survival rate at 3 years was found to be 82%. However, this survival rate should be viewed with caution due to the small sample size (81 patients) but also the specificity of the patients who had the same histological type.

The overall survival at 3 years observed in our cohort was 0.6 [SE = 0.03; CI 95% = 0.54–0.66], which is close to the results found in the literature. In a meta-analysis conducted on individual studies done in Iran, the pooled 3 years survival rate estimated by a random effect model was 0.64 [CI 95% = 0.59–0.7] [31]. This result is very close to the rate found by [37], which is 70.67 [CI 95% = 66.4–74.93] in EMRO countries.

The literature on prognostic factors for CRC is extremely rich [1, 39]. Regarding age, our results reveal a high-risk period after 72 years and a low-risk period before 30 years (Figure 3) . This result is interesting because the majority of studies on CRC predictive factors use age as a categorical variable throughage groups, thus losing an estimate of their prognostic effect at age time points [15, 1, 8].

We also identified factors that are considered to be barriers to health care seeking for CRC patients. Our results reveal that health insurance, pathological stage, and surgery were the main prognostic factors. Age, residency, and site also have a significant impact on the predicted survival rate.

In fact, the health coverage of patients is a condition to their regular access to care and thus have an important impact on their survival probability after diagnosis. The influence of financial barriers affects perceptions of severity, importance, and attribution of symptoms. When patients believe they cannot afford treatment, they may be more likely to downplay the severity of their symptoms [43].

Patients must first detect and interpret CRC symptoms as requiring medical attention before approaching the health system. When this does not happen, it is referred to as an apparaisal delay [43] Assessment delay is defined as the time between when patients first detects their symptoms and when they first disclose them to a health professional. This delay is even more important when patients do not have health insurance and access to care is difficult.

In addition, other factors such as geographical distance and unfavourable economic conditions may also delay diagnosis. In a study done on a large panel of patients, the risk of death increased significantly beyond 31 days from diagnosis to treatment interval [29]. We found that living in a rural area has a negative effect on CRC survival. This is closely related to the large distance between the health care centers and the places of residence in rural areas.

In addition, a prevention strategy based on the individual risk of colorectal cancer should be systematically adopted by health professionals [41]. Primary prevention for the general population is important. However, in order to be more effective, it should be targeted at the high-risk population [17]. Our results show that people aged 30 years or older, living in rural areas, without health insurance, at a distant stage and who have not had surgery, constitute a subgroup of patients with poor prognonsis. Therefore, screening programs should be offered systematically to those patients.

The good predictive performance of RSF is an interesting result, especially in the context of predicting prognostic factors. RSF are flexible and have fewer assumptions about the data in hand. Even though they are considered black-box models, the methodological development of interpretability techniques has made their results more intelligible. Therefore, they are a good alternative model for analyzing survival data for cancer studies [38, 34].

It is necessary to combine several methods to ensure consistency of results. This is the case in our study. We combined conventional methods such as the Kaplan-Meier estimator and the Cox model with the Random Forest survival method and the PDP interpretation approach. The agreement between the methods used confirms the low degree of sensitivity of our results [48].

Our study is the first in Morocco based on an innovative and robust statistical approach. Thus, the sample studied was subjected to an important data quality verification work. Even if our data come from patient files, they have been completed if necessary by an actif follow-up reflecting more precision for the endpoint.

It is clear that our cohort recruited at Hassan II University Hospital cannot claim to be representative of the Moroccan population, but this will be improved in our future studies, where we intend to design a multicenter study to reflect the profile of Moroccan CRC patients as much as possible.

Furthermore, adding data on the genetic profile of the included patients is an excellent way to obtain very revealing results [7].

Also, a clinical decision support platform for colorectal cancer is needed in order to make clinical information easily usable by practitioners. Unfortunately, we are limited by the retrospective nature of our study. Ideally, the physician should have access to such a tool at each suspicious consultation to predict a risk score of developing colorectal cancer in order to perform real-time clinical prevention. Besides, the risk of information bias is not totally discarded, especially for disadvantaged patients with difficulties accessing care.

## Conclusion

Management and control of a complex disease such as colorectal cancer cannot rely only on the classical treatment approach and must take advantage of more integrated predictive and personalized medicine at a policy level. The effectiveness of such an approach can be increased by targeting the preventable nature of the disease and promoting access to care for the most vulnerable individuals through the expansion of health insurance coverage, which is an urgent national need. The generalization of health coverage will make it possible to reduce the risk of cancer by encouraging early detection and by reducing the out-of-pocket expenses which penalize in priority the individuals who have a low standard of living.

## Figures and Tables

**Table 1:** Socio-demographic characteristics of CRC patients

	Female (N=181)	Male (N=165)	Overall (N=346)

**Age (years)**			
Mean (SD)	56.5 (13.4)	58.3 (15.3)	57.3 (14.3)
Median [Min, Max]	57.0 [23.0, 82.0]	59.0 [19.0, 89.0]	58.0 [19.0, 89.0]
**Insurance**			
Yes	162 (89.5%)	151 (91.5%)	313 (90.5%)
No	19 (10.5%)	14 (8.5%)	33 (9.5%)
**Residency**			
Urban	146 (80.7%)	133 (80.6%)	279 (80.6%)
Rural	35 (19.3%)	32 (19.4%)	67 (19.4%)

**Table 2 :** Clinical characteristics of CRC patients

	Colon (N=186)	Rectum (N=160)	Overall (N=346)

**Delay to start treatment (days)**			
Mean (SD)	36.5 (51.2)	71.4 (72.8)	52.7 (64.4)
Median [Min, Max]	21.0 [0, 281]	53.0 [0, 503]	34.0 [0, 503]
**Personal history**			
No	179 (96.2%)	157 (98.1%)	336 (97.1%)
Yes	7 (3.8%)	3 (1.9%)	10 (2.9%)
**Tumor Differentiation**			
Undifferentiated	15 (8.1%)	11 (6.9%)	26 (7.5%)
Moderately differentiated	66 (35.5%)	51 (31.9%)	117 (33.8%)
Well differentiated	105 (56.5%)	98 (61.3%)	203 (58.7%)
**Tumor subsite**			
Inferior rectum	--	84 (52.5%)	84 (24.3%)
Left colon	143 (76.9%)	--	143 (41.3%)
Middle rectum	--	58 (36.3%)	58 (16.8%)
Right colon	34 (18.3%)	--	34 (9.8%)
Superior rectal	--	18 (11.3%)	18 (5.2%)
Transverse colon	9 (4.8%)	0 (0%)	9 (2.6%)
**Stage**			
Local	52 (28.0%)	49 (30.6%)	101 (29.2%)
Regional	33 (17.7%)	23 (14.4%)	56 (16.2%)
Distant	101 (54.3%)	88 (55.0%)	189 (54.6%)
**Histological type**			
Mucinous	165 (88.7%)	148 (92.5%)	313 (90.5%)
Adenocarcinoma	17 (9.1%)	7 (4.4%)	24 (6.9%)
Signet ring cell	4 (2.2%)	5 (3.1%)	9 (2.6%)
**Surgery**			
Yes	97 (52.2%)	88 (55.0%)	185 (53.5%)
No	89 (47.8%)	72 (45.0%)	161 (46.5%)
**MSI/MSS Status**			
No	166 (89.2%)	156 (97.5%)	322 (93.1%)
Yes	20 (10.8%)	4 (2.5%)	24 (6.9%)
**RAS mutated**			
No	179 (96.2%)	157 (98.1%)	336 (97.1%)
Yes	7 (3.8%)	3 (1.9%)	10 (2.9%)

**Table 3 :** Overall survival rates at 1 year, 2 years and 3 years

Year	Survival rates	SE	95% CI-lower	95% CI-upper

1 year	0.87	0.02	0.84	0.91
2 years	0.77	0.02	0.73	0.82
3 years	0.60	0.03	0.54	0.66

**Table 4 :** Univariate and multivariate Cox regression model

Variables		all	HR (univariable)	HR (multivariable)

Sex	Female	181 (52.3)	-	-
	Male	165 (47.7)	1.67 (1.13–2.47, p=0.010)	1.46 (0.98–2.20, p=0.066)
Age	Mean (SD)	57.3 (14.3)	1.02 (1.00–1.03, p=0.036)	1.02 (1.00–1.03, p=0.031)
Delay	Mean (SD)	52.7 (64.4)	1.00 (1.00–1.00, p=0.990)	1.00 (0.99–1.00, p=0.161)
Insurance	No	33 (9.5)	-	-
	Yes	313 (90.5)	0.44 (0.27–0.72, p=0.001)	0.35 (0.20–0.61, p<0.001)
Residency	Rural	67 (19.4)	-	-
	Urban	279 (80.6)	0.58 (0.38–0.89, p=0.012)	0.53 (0.34–0.85, p=0.008)
Personal.history	No	336 (97.1)	-	-
	Yes	10 (2.9)	0.63 (0.15–2.54, p=0.511)	1.08 (0.26–4.57, p=0.914)
Site	Colon	186 (53.8)	-	-
	Rectum	160 (46.2)	1.23 (0.83–1.81, p=0.296)	1.86 (1.20–2.87, p=0.005)
Stage	Distant	189 (54.6)	-	-
	Local	101 (29.2)	0.08 (0.03–0.16, p<0.001)	0.15 (0.06–0.36, p<0.001)
	Regional	56 (16.2)	0.16 (0.08–0.33, p<0.001)	0.31 (0.13–0.74, p=0.008)
Tumor differentiation	Undifferentiated	26 (7.5)	-	-
	Moderately differentiated	117 (33.8)	0.81 (0.41–1.62, p=0.555)	0.61 (0.25–1.48, p=0.272)
	Well differentiated	203 (58.7)	0.52 (0.26–1.02, p=0.057)	0.53 (0.22–1.27, p=0.155)
Histological type	Mucinous	313 (90.5)	-	-
	Adenocarcinoma	24 (6.9)	0.76 (0.33–1.74, p=0.522)	1.11 (0.44–2.78, p=0.825)
	Signet ring cell	9 (2.6)	2.14 (0.68–6.78, p=0.196)	2.20 (0.53–9.09, p=0.278)
Surgery	No	161 (46.5)	-	-
	Yes	185 (53.5)	0.16 (0.11–0.25, p<0.001)	0.31 (0.18–0.55, p<0.001)
MSI.MSS status	No	322 (93.1)	-	-
	Yes	24 (6.9)	0.68 (0.30–1.54, p=0.353)	1.05 (0.43–2.57, p=0.922)
Ras mutated	No	336 (97.1)	-	-
	Yes	10 (2.9)	1.52 (0.62–3.73, p=0.362)	0.82 (0.33–2.08, p=0.680)

## Data Availability

The dataset analysed during the current study is available in the figshare repository, accessible via this link: https://figshare.com/articles/dataset/dataset_CRC_Fes_csv/21821439
